# Understanding Online Health Information Consumption Through Web Analytics of the Italian Society of Pharmacology Magazine: 3-Year Descriptive Analysis

**DOI:** 10.2196/93245

**Published:** 2026-05-05

**Authors:** Agnese Graziosi, Michele Santoni, Luigi Cari, Lucia Gozzo, Stefania Crucitta, Antonella Di Sotto, Concetta Altamura, Silvia Di Giacomo, Laura Rizzi, Chiara Ruocco, Alessandra Bitto, Gianni Sava

**Affiliations:** 1 Department of Medical Biotechnology and Translational Medicine University of Milan Milano, Lombardy Italy; 2 Department of Biomedical Sciences University of Cagliari Cagliari, Sardinia Italy; 3 Department of Medicine and Surgery University of Perugia Perugia, Umbria Italy; 4 Clinical Trial Unit Policlinico G.Rodolico San Marco Catania, Sicily Italy; 5 Chemical-Clinical Analyses Unit Azienda Socio Sanitaria Territoriale Grande Ospedale Metropolitano Niguarda Milano, Lombardy Italy; 6 Department of Physiology and Pharmacology “V. Erspamer" Sapienza University of Rome Roma, Lazio Italy; 7 Department of Precision and Regenerative Medicine and Ionian Area University of Bari Aldo Moro Bari, Apulia Italy; 8 Department of Food Safety, Nutrition and Veterinary Public Health Istituto Superiore di Sanità Roma, Lazio Italy; 9 Department of Medicine and Surgery University of Milano-Bicocca Milano, Lombardy Italy; 10 Department of Biomedical Sciences, Dental Sciences, and Morpho-Functional Imaging University of Messina Messina, Sicily Italy; 11 Italian Society of Pharmacology Milano, Lombardy Italy

**Keywords:** digital scientific communication, web analytics, health information, information dissemination, pharmacology

## Abstract

**Background:**

The COVID-19 pandemic underscored that access to reliable and expert-driven scientific information is not only essential but also lifesaving. Since 2020, the Italian Society of Pharmacology has been publishing *SIF Magazine*, an online magazine dedicated to citizens. This journal was created to make pharmacology accessible to the public, highlighting its impact on health and quality of life while clarifying the truths, theories, and misconceptions surrounding drugs and their use.

**Objective:**

This work analyzed web interaction data from *SIF Magazine* to understand how the public reaches and engages with an online scientific journal and gather practical insights for improving digital scientific communication.

**Methods:**

The data analyzed in this study were obtained from the web analytics of the *SIF Magazine* website. The analysis covers 3 years (2022-2024). By studying patterns of access, navigation, and engagement, the analysis clarified which types of scientific content connect most with users, how people find and choose trustworthy sources, and what they do after reaching them.

**Results:**

Average monthly site visits increased from 120,024 in the partial period examined in 2022 to 128,059 in 2023 and 200,379 in 2024, paralleled by higher monthly views (155,785 in 2022, 165,438 in 2023, and 254,297 in 2024). The engagement rate declined modestly (36% in late 2022, 35% in 2023, and 29% in 2024), consistent with scale-related dilution from an expanding top-of-funnel audience. Category-level analyses of top-performing articles indicated disproportionate interest in renal, urogenital, and sexual disorders followed by inflammation and pain and gastrointestinal diseases. Seasonal analyses showed recurrent peaks for season-linked topics (eg, motion sickness, photosensitivity reactions, and influenza vaccination) during expected periods.

**Conclusions:**

Together, these findings underscore the importance of data-driven content planning and continuous performance monitoring to sustain the effectiveness of digital scientific communication platforms.

## Introduction

The COVID-19 pandemic underscored that access to reliable and expert-driven scientific information is crucial. In those years of uncertainty, the spread of misinformation and fake news, particularly concerning drugs, vaccines, and therapeutic strategies, posed a serious threat to public health [1,2]. Accordingly, a recent editorial published in *The Lancet* has appropriately drawn attention to misinformation and disinformation as major threats to public health [3]. Since 2020, the Italian Society of Pharmacology has been publishing *SIF Magazine* [4], an online magazine dedicated to citizens in which Italian Society of Pharmacology experts clarify pharmacological topics of medical and scientific interest, providing clear and useful information. This journal was created to make pharmacology accessible to the public, highlighting its impact on health and quality of life while clarifying the truths, theories, and misconceptions surrounding drugs and their use.

Citizen participation in medical-scientific discussions is essential for an informed health care ecosystem, yet it can only be meaningful when grounded in accurate and unbiased information [5]. In today’s digital landscape, how individuals search for; access; and, ultimately, internalize scientific content is evolving at an unprecedented pace [6]. This rapid transformation, driven by the proliferation of online platforms and artificial intelligence, the speed of information dissemination, and the growing visibility of scientific debate in everyday life, reshapes not only how people encounter medical knowledge but also how they form opinions, make health-related decisions, and engage with experts [7]. Therefore, understanding these dynamics is critical. It allows us to identify barriers to comprehension, highlight factors that promote trust or distrust, and design communication strategies that enable citizens to navigate complex scientific information more confidently and responsibly [8]. However, few studies have systematically analyzed real-world user behavior on society-driven scientific communication platforms.

To address this gap, we conducted a 3-year descriptive analysis of web interaction data from *SIF Magazine*. This study aimed to characterize user behavior and provide actionable insights to optimize digital scientific communication strategies.

## Methods

This study was reported in accordance with the STROBE (Strengthening the Reporting of Observational Studies in Epidemiology) guidelines adapted to the descriptive nature of web analytics data.

### Data Source and Period of Analysis

The data were obtained from *SIF Magazine* web analytics and cover 2022 to 2024, with 2022 representing a partial period (July 11, 2022-December 31, 2022).

### Analysis of Overall Site Performance

Overall site performance was evaluated using key web interaction metrics, including traffic (total visits and views), interactions (total clicks and downloads), engagement rate, and user characteristics (location, acquisition channel, and device type). Engagement rate was defined as the percentage of engaged sessions, where an engaged session refers to a visit lasting more than 10 seconds, including at least 2 page views or triggering a conversion event. Conversion events represent predefined user actions considered meaningful for the website’s objectives, such as downloads or other key interactions with the content.

### Keyword Analysis

Journal performance was evaluated by analyzing the search keywords leading users to *SIF Magazine* as a measure of search engine optimization (SEO) effectiveness. Metrics included URL clicks (number of visits generated by each query); impressions (number of times the URL appeared in search results); and click-through rate (CTR; clicks/impressions × 100), which reflects the ability of each query to convert visibility into user visits.

### Analysis of the Most Viewed Documents

To identify the content generating the greatest reader interest, we analyzed documents with at least 1000 views during follow-up.

### Analysis of the Most Viewed Articles

As articles represent the main content category, we analyzed the 15 most viewed articles. Because publication dates differed, views were normalized by months of online availability to allow for accurate comparison.

### Seasonal Analysis

Seasonal trends in reader interest were assessed by analyzing the monthly distribution of views for articles with season-related topics and at least 20 months of follow-up. Articles were classified as seasonal if their topic was expected to generate higher interest during a specific period of the year (eg, respiratory infections, seasonal allergies, or travel-related drugs).

### Data Analysis

Collected data were analyzed descriptively to identify trends over time, including year-to-year comparisons of metrics, user distribution across categories, and ranking of content by page views to assess changes in audience size, access methods, engagement, and content preferences.

### Ethical Considerations

This study was based exclusively on aggregated and anonymized web analytics data with no involvement of human participants or identifiable personal data. According to institutional and national regulations [9-11], ethics committee approval was not required for this type of study.

## Results

### Overall Site Performance

Overall site performance was analyzed across 4 domains: traffic, interactions, engagement rate, and user characteristics.

### Traffic (Visits and Views)

The overall performance of the *SIF Magazine* website showed significant growth from 2022 to 2024 (Tables 1-3). Average monthly site visits increased from 120,024 in 2022 to 200,379 in 2024, corresponding to an increase of 80,355 visits (+66.9%). Similarly, average monthly total views rose from 155,785 to 254,297, representing an increase of 98,512 views (+63.2%).

**Table 1:** Data regarding site visits and views.

Year	Site visits per month, mean	Total views per month, mean
2022 (July 11-December 31)	120,024	155,785
2023	128,059	165,438
2024	200,379	254,297

**Table 2:** Data related to the interaction types from users.

Year	Total clicks, n	Total downloads, n
2022 (July 11-December 31)	747	238
2023	2046	464
2024	5264	1136

**Table 3:** Data regarding the engagement rate of SIF Magazine over the period investigated.

Year	Engagement rate (%)
2022 (July 11-December 31)	36
2023	35
2024	29

### Interactions (Clicks and Downloads)

User interactions increased markedly over time. Total clicks, defined as cumulative interactions with on-page elements (eg, links, buttons, and navigation items), increased more than 6-fold from 2022 to 2024. Downloads also increased from 238 in 2022 to 1136 in 2024, reflecting growing user engagement with downloadable content.

### Engagement Rate

Despite the overall rise in traffic and interactions, the engagement rate slightly declined from 36% in late 2022 to 29% in 2024 (Table 3). This trend is likely attributable to the increasing proportion of new or casual visitors associated with platform growth.

### User Characteristics

As *SIF Magazine* is published in Italian, readership is mainly concentrated in Italy, with the highest activity in major urban areas and a small but growing international presence. Milan and Rome consistently showed the highest user engagement, followed by Bologna, Naples, and Turin. The 2024 data, providing a more detailed geographic breakdown, confirmed this pattern and highlighted a gradual expansion of the audience beyond national borders.

User access patterns showed that organic search was the dominant acquisition channel across all years, generating over 90% (80,824/86,549, 93.4% in 2022; 599,269/637,813, 93.9% in 2023; and 2,222,054/2,405,541, 92.4% in 2024) of the total traffic (Table 4), reflecting strong search engine visibility and content relevance. Direct traffic was the second-largest source, indicating growing brand recognition and recurring readership. Regarding device use, mobile devices were the primary access mode, accounting for more than 80% (86,725/103,319, 83.9% in 2022; 693,704/814,202, 85.2% in 2023; and 2,475,706/3,050,299, 81.2% in 2024) of all views over the 3-year period (Table 5), whereas desktop use showed a modest increase and tablet traffic remained marginal. These findings highlight the importance of a strong search engine presence and mobile-first optimization.

**Table 4:** User distribution across channels per year.

Channel type	Users acquired in 2022, n	Users acquired in 2023, n	Users acquired in 2024, n
Organic search	80,824	599,269	2,222,054
Direct traffic	5582	37,237	174,135
Organic social (visits generated from unpaid posts or shares on social media platforms)	34	266	5916
Unassigned	46	543	1193
Referral	61	494	2222
Organic shopping (visits originating from non-paid product listings on shopping platforms or marketplaces)	1	4	12
Email	0	0	6
Organic video (visits coming from unpaid video content sources, typically including platforms, such as YouTube or embedded video links, where the traffic is not driven by paid promotion)	1	0	3

**Table 5:** User and type of device per year.

Device category	Users acquired in 2022, n	Users acquired in 2023, n	Users acquired in 2024, n
Mobile	86,725	693,704	2,475,706
Desktop	14,733	118,826	521,258
Tablet	1858	1654	53,223
Smart television	3	18	112

### Keyword Analysis

Each keyword represents a search term used by users to reach *SIF Magazine* through search engines. Analysis of these data provides insights into which queries drive traffic and how organic visibility evolves over time.

The first step of the analysis focused on the top 10 queries from the beginning of performance monitoring to the time of our analysis (Table 6).

**Table 6:** Performance ranking of the top 10 search queries by URL clicks, with the corresponding impressions and click-through rate (CTR).

Keyword^a^ rank based on URL clicks	Query^a^	CTR, URL clicks/impressions (%)
1	“St. John’s Wort”	14,251/325,306 (4.4)
2	“Triptorelin”	11,996/69,530 (17.3)
3	“*Serenoa repens*”	8908/161,854 (5.5)
4	“Romosozumab”	8440/35,865 (23.5)
5	“Rheumatoid arthritis can be cured”	7964/102,405 (7.8)
6	“St. John’s wort properties”	7770/53,573 (14.5)
7	“Aloe vera benefits”	7215/90,228 (8)
8	“Milk thistle”	6666/301,108 (2.2)
9	“Ginseng”	6152/517,381 (1.2)
10	“*Helicobacter pylori* definitive cure 2023”	6053/26,446 (22.9)

^a^“Queries” and “keywords” are used interchangeably to indicate search terms entered by users.

### User Search Behavior: Analysis of Top Queries and CTR

From July 11, 2022, to December 31, 2024, St John’s wort (*Hypericum perforatum L*) ranked first with a CTR of 4.4% (14,251 URL clicks/325,306 impressions). The related query “St. John’s wort properties” showed a higher CTR (7770 URL clicks/53,573 impressions, 14.5%). “Triptorelin” ranked second with a CTR of 17.3% (11,996 URL clicks/69,530 impressions), whereas “*Serenoa repens*” generated a CTR of 5.5% (8908 URL clicks/161,854 impressions). “Romosozumab” showed one of the highest CTRs (8440 URL clicks/35,865 impressions, 23.5%). “Rheumatoid arthritis can be cured” achieved a CTR of 7.8% (7964 URL clicks/102,405 impressions), and “Aloe vera benefits” achieved a CTR of 8.0% (7215 URL clicks/90,228 impressions). In contrast, “Milk thistle” and “Ginseng” showed lower CTRs (6666 URL clicks/301,108 impressions, 2.2% and 6152 URL clicks/517,381 impressions, 1.2%, respectively) despite high impression counts. “*Helicobacter pylori* definitive cure 2023,” although last by URL clicks, showed a high CTR of 22.9% (6053 URL clicks/26,446 impressions). Normalization of CTR by days of availability substantially modified rankings (Table 7). “Romosozumab” ranked first, followed by “*Helicobacter pylori* definitive cure 2023” and “Triptorelin,” whereas “St. John’s Wort” moved to fourth place. “Rheumatoid arthritis can be cured” and “Aloe vera benefits” showed no change, whereas other queries exhibited minor ranking shifts. This normalization accounts for differences in exposure time and highlights highly impactful content independent of publication date.

**Table 7:** Ranking of search queries based on normalized click-through rate (CTR) by days of availability.

Normalized rank	Query	Publication date	Days of availability, n	Normalized CTR (%)	Normalized CTR/days	Nonnormalized rank
1	“Romosozumab”	September 28, 2023	461	23.53	0.05104	4
2	“*Helicobacter pylori* definitive cure 2023”	June 8, 2023	573	22.89	0.03995	10
3	“Triptorelin”	February 16, 2023	685	17.25	0.02518	2
4	“St. John’s wort properties”	July 28, 2022	888	14.5	0.01633	6
5	“Rheumatoid arthritis can be cured”	November 17, 2022	776	7.78	0.01003	5
6	“*Serenoa repens*”	May 25, 2023	587	5.5	0.00937	3
7	“Aloe vera benefits”	June 24, 2021	1287	8	0.00622	7
8	“St. John’s Wort”	July 28, 2022	888	4.38	0.00493	1
9	“Milk thistle”	April 27, 2023	615	2.21	0.00359	8

A year-by-year analysis based on URL clicks compared the top queries in 2023 and 2024 (Table 8). “Triptorelin” ranked first in 2023 and remained second in 2024, confirming sustained interest. “St. John’s Wort” rose from fourth place in 2023 to first place in 2024 together with “St. John’s Wort properties.” In contrast, COVID-19–related queries such as “Brufen covid” and “Brufen 600 covid” disappeared from the 2024 ranking. “Romosuzumab” improved from seventh place in 2023 to third place in 2024, whereas new entries in 2024 included “*Cimicifuga*” and the return of “Minoxidil.” Overall, comparison between 2023 and 2024 highlights shifts in user interests, sustained relevance of selected topics, and the need for continuous SEO strategy adaptation.

**Table 8:** The top 10 queries for the years 2023 and 2024 based on URL clicks.

Rank based on URL clicks	Keywords in 2023	Keywords in 2024
1	“Triptorelin”	“St. John’s Wort”
2	“Pharmacological treatments for *Helicobacter pylori*”	“Triptorelin”
3	“Brufen covid”	“Romosuzumab”
4	“St. John’s Wort”	“*Serenoa* *repens*”
5	“Mandatory vaccines”	“St. John’s Wort properties”
6	“Brufen 600 covid”	“Rheumatoid arthritis”
7	“Romosuzumab”	“Aloe vera benefits”
8	“Minoxidil”	“Milk thistle”
9	“Antibiotics for children”	“Cimicifuga”
10	“Brufen covid”	“Minoxidil”

### Analysis of the Most Viewed Documents

To identify the content generating the greatest reader interest, we analyzed documents with at least 1000 views during follow-up. This included 65.6% (241/368) of the selected articles, 31.5% (116/368) of the support entries, and 3% (11/368) of the frequently asked questions.

### Analysis of the Most Viewed Articles

As articles represented the main content category, we analyzed the 15 most viewed articles. Because publication dates differed, views were normalized by months of online availability to allow for accurate comparison. As shown in Table 9, the normalized top 15 articles achieved approximately 80,000 views per month, with a mean of 5297 views per article and a mean engagement time of 67.1 seconds.

**Table 9:** Most viewed articles in the reference period.

	Total visualizations, n	Visualizations, mean	Time of involvement (s), mean
Article details	1,942,262	129,484	75.1
Normalized article details	79,453 (per month)	5297 (per month)	67.1

### Study of Category Distribution

We analyzed the thematic categories of the top 15 articles to determine whether certain topics were associated with higher reader interest (Table 10).

**Figure 1:**
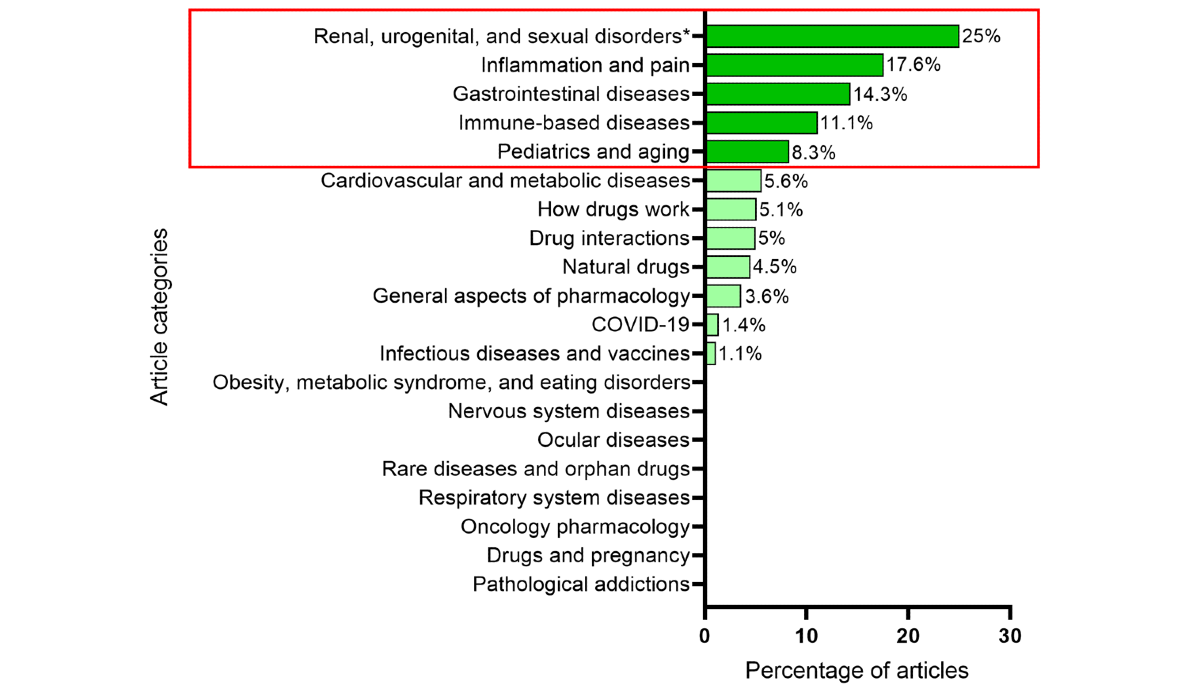
Categories with the highest proportion of articles in the top 15. *Only 4 articles published.

To correct for potential bias due to unequal category representation, a second normalization was applied based on the total number of articles published per category. For each category, we calculated the percentage of articles appearing in the top 15. As shown in Figure 1, “Renal, urogenital, and sexual disorders” showed the highest proportion of top 15 articles, followed by “Inflammation and pain” and “Gastrointestinal diseases,” indicating that these thematic areas attract greater reader interest independent of category size.

**Table 10:** Categories of the top 15 articles (n=19).

Article category	Articles, n (%)
Inflammation and pain	3 (15.8)
Natural medicines	3 (15.8)
Cardiovascular and metabolic diseases	2 (10.5)
How drugs work	2 (10.5)
Pediatrics and aging	2 (10.5)
Other categories	7 (36.9)

### Most Popular Articles in 2023 and 2024

Analysis of the most viewed articles in 2023 and 2024 showed consistent reader interest in inflammation, chronic pharmacological management, and natural products, with a shift in emphasis between years. In 2023, engagement was strongly influenced by the COVID-19 pandemic, with the leading article focused on anti-inflammatory drug use during SARS-CoV-2 infection. Other prominent topics included inflammatory bowel syndrome, gout arthritis, and St. John’s wort, reflecting a focus on infection-related inflammation and pharmacological safety. In 2024, interests were more diversified. Although articles on gout arthritis and expired medication management remained highly viewed, interest in pandemic-related topics declined, whereas engagement increased for general pharmacological and holistic health care themes, including thyroid dysfunction, dietary supplementation, and rheumatoid arthritis.

### Seasonal Trends Across the Topic Area

**Figure 2:**
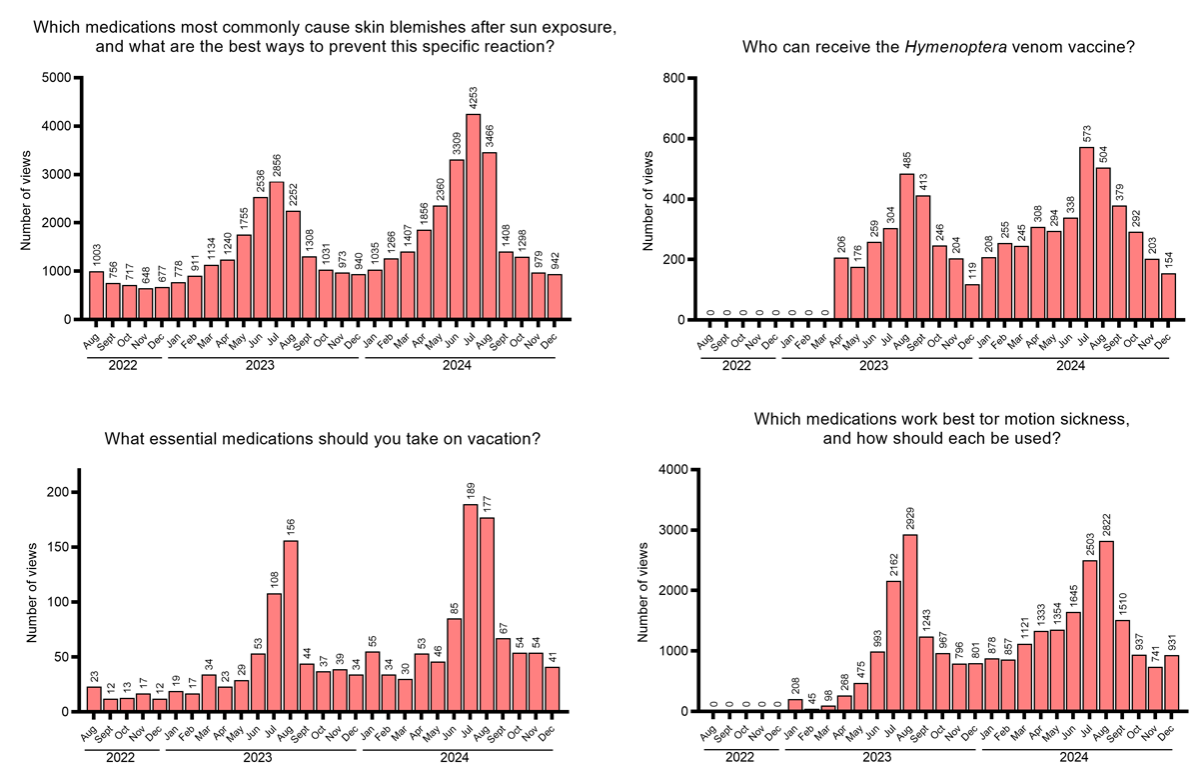
Trend of views over time for seasonal articles (higher in summer).

**Figure 3:**
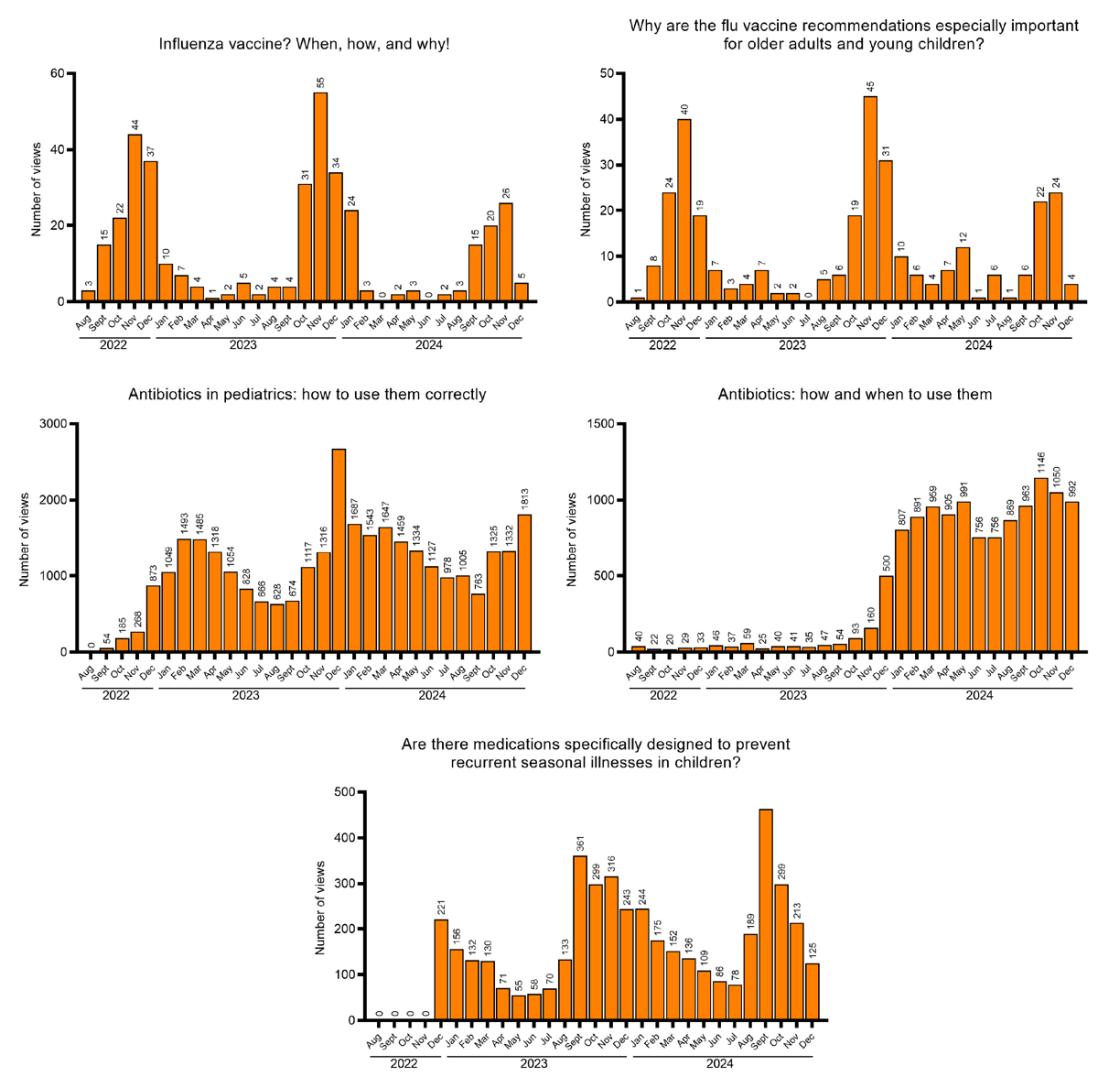
Trend of views over time for seasonal articles (higher in autumn-winter).

**Figure 4:**
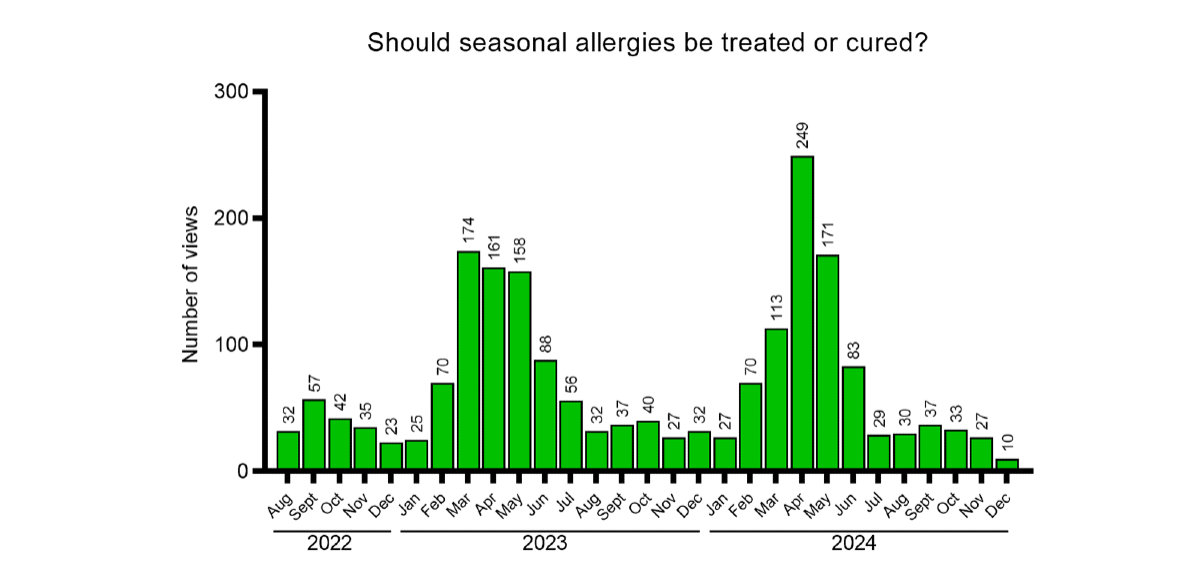
Trend of views over time for seasonal articles (higher in spring).

**Figure 5:**
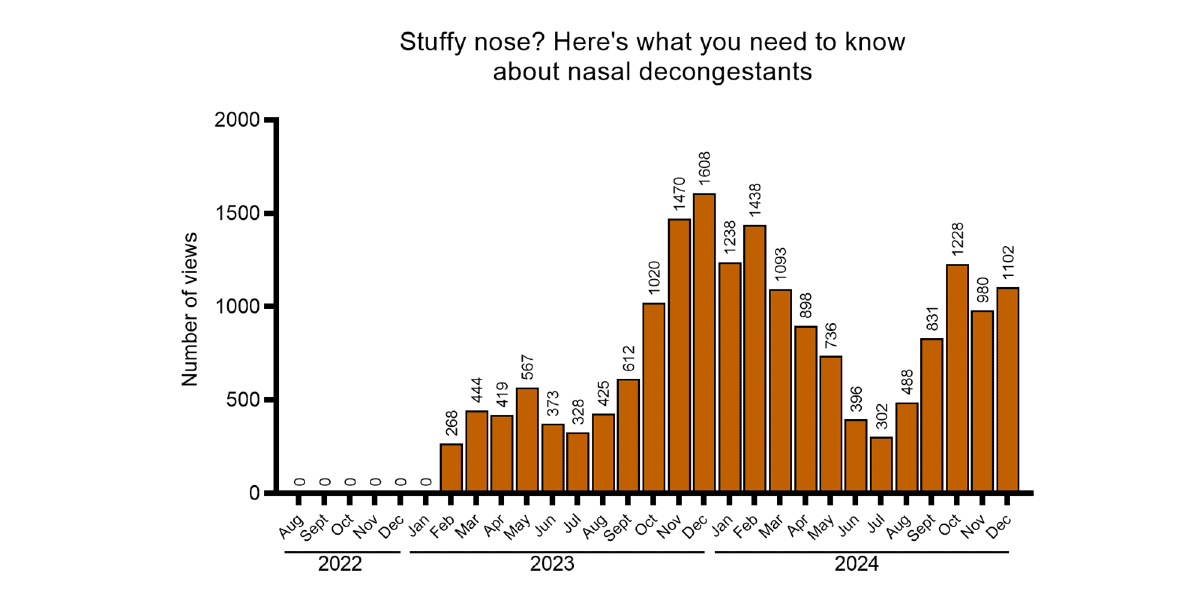
Trend of views over time for seasonal articles (lower in summer).

Among all documents published in the reference period, 11 articles met the inclusion criteria specified in the Methods section: 4 (36.4%) with potentially higher summer interest (sun-related skin reactions, *Hymenoptera* venom vaccine, vacation medications, and motion sickness; Figure 2), 5 (45.5%) with potentially higher autumn-winter interest (influenza vaccine, influenza vaccination in older adults and children, antibiotic use in adults and children, and prevention of recurrent seasonal illnesses in children; Figure 3), 1 (9.1%) with potentially higher spring interest (seasonal allergies; Figure 4), and 1 (9.1%) with potentially lower summer interest (nasal decongestants; Figure 5).

## Discussion

### Principal Findings

The 3-year analysis of *SIF Magazine* web analytics data shows significant growth in user engagement. Total site visits increased by 66.9%, whereas page views and clicks grew roughly 6-fold compared to the baseline year. Downloads of materials nearly quadrupled, indicating active use of the available resources. Organic search was the dominant acquisition channel, whereas direct traffic and social referrals played a smaller role, suggesting strong search engine visibility and rising brand recognition. Mobile devices accounted for most access, underscoring the importance of mobile-friendly design. Topic-wise, content on natural products, chronic diseases, and symptom-oriented issues attracted the most attention, whereas interest in COVID-19–related topics declined markedly in 2024. Together, these findings indicate that *SIF Magazine*’s audience has expanded substantially in size and engagement, with evolving information needs.

### Transformation of Health Information–Seeking Behavior

In the past, individuals have primarily relied on health care professionals, printed materials, and personal networks for health information [12]. The emergence of information and communications technologies has profoundly transformed these behaviors, making the internet one of the main sources of medical information and services [13].

While this transition offers clear advantages, including convenience, anonymity, and greater autonomy, it also exposes users to inaccurate or misleading content, with potential consequences including harmful self-diagnosis, inappropriate self-treatment, reduced adherence to medical advice, and financial losses [14]. In this context, the dissemination of accurate, evidence-based information by qualified experts, which is central to *SIF Magazine*’s mission, plays a crucial role in supporting citizens and mitigating the risks associated with unreliable online content.

### Interpretation of Engagement Trends

Therefore, continuous evaluation of performance metrics is essential to refine communication strategies and ensure responsiveness to public needs. The analysis of *SIF Magazine*’s web performance from 2022 to 2024 provides insights into user engagement with scientific digital content and illustrates the interaction among content, user experience, and external factors. The growth in traffic and engagement observed in this study aligns with previous research showing increasing demand for accessible and trustworthy online health information [15-18], a trend further amplified after the COVID-19 pandemic [19]. The marked rise in site visits and views demonstrates expanding reach, whereas the substantial increase in clicks and downloads indicates active user interaction and perceived value of the content. These trends may reflect increased demand for online health information and a broader shift in user behavior toward more structured and goal-oriented information-seeking strategies. This evolution is likely driven by improvements in digital health literacy and by the widespread use of algorithm-driven search systems, which facilitate rapid access to targeted information but may also shape user exposure through ranking mechanisms. Consistent with prior findings, users are more likely to engage deeply with scientific content when it is relevant, accessible, and easy to navigate [20]. Although the engagement rate showed a slight decline (from 36% to 29%), this is likely attributable to the influx of new and more casual users accompanying platform growth rather than a true reduction in interest. The predominantly Italian readership is consistent with the language of publication, whereas the emergence of an international audience suggests opportunities for future expansion.

### Content Preferences and Temporal Dynamics

The thematic patterns identified in this analysis highlight evolving public interests. The sustained attention to natural products and chronic disease management reflects a growing emphasis on self-care and long-term health management strategies [20]. At the same time, the observed seasonal peaks in specific articles confirm that user engagement is influenced by predictable fluctuations in public health concerns, reinforcing the importance of timely and context-aware content planning. Moreover, the analysis of the most viewed documents confirms that articles are the primary content drivers, with thematic areas such as “Renal, urogenital, and sexual disorders,” “Inflammation and pain,” and “Gastrointestinal diseases” attracting disproportionate attention.

The comparison between 2023 and 2024 reveals a clear decline in COVID-19–related queries (eg, “brufen covid” and “brufen 600 covid”) and a shift toward broader pharmacological and chronic disease–related topics. This trend is consistent with the progressive normalization of the postpandemic context and has been observed in other studies analyzing online health information–seeking behavior [19]. These findings emphasize the need for dynamic editorial strategies capable of adapting to changing public interests.

### Implications for Digital Scientific Communication

From a strategic perspective, the predominance of organic search highlights the importance of SEO in ensuring content visibility, whereas the presence of direct traffic suggests increasing brand recognition and user loyalty. The high proportion of mobile access further underscores the necessity of adopting a mobile-first design to optimize user experience. More broadly, this study demonstrates how web analytics can function as a powerful tool for guiding scientific communication. By identifying high-performing topics, user preferences, and access patterns, scientific societies can optimize content development, improve dissemination strategies, and better align their output with public information needs. In an increasingly competitive and information-saturated digital environment, such data-driven approaches are essential to maximize impact and relevance [21].

### Limitations

Several limitations should be acknowledged. First, this analysis is based on aggregated web analytics data and does not capture individual-level characteristics such as user demographics, motivations, or comprehension. Metrics such as page views, clicks, and downloads provide indirect indicators of engagement but do not necessarily reflect knowledge acquisition or behavior change. Second, web traffic is influenced by external factors beyond content quality, including search engine algorithm updates, media coverage, and concurrent public health campaigns, which were not controlled for in this study. Third, the analysis focuses on a single Italian-language platform, which may limit the generalizability of the findings to other contexts, populations, or types of digital health communication. Finally, while keyword and content categorization provide useful insights, they may oversimplify complex user interests and information needs. Future studies integrating web analytics with qualitative approaches such as surveys or interviews could provide a more comprehensive understanding of user behavior and information-seeking motivations.

### Conclusions

This study demonstrates that a scientific society-driven online platform such as *SIF Magazine* can achieve substantial reach and engagement when delivering evidence-based, accessible, and timely content. The findings highlight the growing public demand for reliable pharmacological information and underscore the importance of adapting communication strategies to evolving user needs. Web analytics should be considered a strategic tool for digital public health communication, enabling continuous monitoring of audience behavior, optimization of content distribution, and early identification of emerging health issues. The integration of data-driven insights into editorial planning can improve the effectiveness, relevance, and impact of scientific dissemination.

In an era increasingly characterized by artificial intelligence, the role of expert-authored, evidence-based content becomes even more critical. Scientific societies have a fundamental responsibility in ensuring that accurate and trustworthy information remains accessible to the public, complementing and balancing automated information retrieval systems.

## Abbreviations

CTR: click-through rate
SEO: search engine optimization
STROBE: Strengthening the Reporting of Observational Studies in Epidemiology

## Data Availability

The data that support this study are available from the corresponding author on reasonable request.
